# Root-associated bacteria modulate the specialised metabolome of *Lithospermum officinale* L.

**DOI:** 10.3389/fpls.2022.908669

**Published:** 2022-08-30

**Authors:** Alicia Varela Alonso, Henry D. Naranjo, Angélique Rat, Nebojša Rodić, Christina I. Nannou, Dimitra A. Lambropoulou, Andreana N. Assimopoulou, Stéphane Declerck, Philipp Rödel, Carolin Schneider, Anne Willems

**Affiliations:** ^1^Institut für Pflanzenkultur GmbH & Co. KG., Schnega, Germany; ^2^Laboratory of Mycology, Earth and Life Institute, Université catholique de Louvain, Louvain-la-Neuve, Belgium; ^3^Laboratory of Microbiology, Department of Biochemistry and Microbiology, Ghent University, Ghent, Belgium; ^4^Laboratory of Organic Chemistry, School of Chemical Engineering, Aristotle University of Thessaloniki, Thessaloniki, Greece; ^5^Natural Products Research Center of Excellence (NatPro-AUTh), Center for Interdisciplinary Research and Innovation (CIRI-AUTh), Thessaloniki, Greece; ^6^Center for Interdisciplinary Research and Innovation (CIRI-AUTh), Balkan Center, Thessaloniki, Greece; ^7^Laboratory of Environmental Pollution Control, Department of Chemistry, Aristotle University of Thessaloniki, Thessaloniki, Greece

**Keywords:** bacteria, *Lithospermum officinale*, metabolism regulation, PGT, secondary metabolites, shikimate pathway, shikonin

## Abstract

Bacteria influence plant growth and development and therefore are attractive resources for applications in agriculture. However, little is known about the impact of these microorganisms on secondary metabolite (SM) production by medicinal plants. Here we assessed, for the first time, the effects of bacteria on the modulation of SM production in the medicinal plant *Lithospermum officinale* (Boraginaceae family) with a focus on the naphthoquinones alkannin/shikonin and their derivatives (A/Sd). The study was conducted in an *in vitro* cultivation system developed for that purpose, as well as in a greenhouse. Targeted and non-targeted metabolomics were performed, and expression of the gene *PGT* encoding for a key enzyme in the A/S biosynthesis pathway was evaluated with qPCR. Three strains, *Chitinophaga* sp. R-73072, *Xanthomonas* sp. R-73098 and *Pseudomonas* sp. R-71838 induced a significant increase of A/Sd in *L. officinale* in both systems, demonstrating the strength of our approach for screening A/Sd-inducing bacteria. The bacterial treatments altered other plant metabolites derived from the shikimate pathway as well. Our results demonstrate that bacteria influence the biosynthesis of A/Sd and interact with different metabolic pathways. This work highlights the potential of bacteria to increase the production of SM in medicinal plants and reveals new patterns in the metabolome regulation of *L. officinale*.

## Introduction

Medicinal plants are commonly defined as plants possessing therapeutic properties or exerting beneficial pharmacological effects on humans or animals (Sofowora et al., 2013; Ahmad et al., 2019). They produce secondary metabolites (SMs) with antioxidant, antimicrobial and/or anti-cancer properties and are thus often used in traditional medicine (Wen et al., 2012; Ahmad et al., 2019).

*Lithospermum officinale*, also known as European stone-seed, is a rhizomatous perennial herb that can reach 90 cm in height. This species belongs to one of the four largest genera, next to *Onosma* spp., *Echium* spp. and *Alkanna* spp., of the Lithospermaceae tribe within the Boraginaceae family (Chacón et al., 2019). It grows in open grasslands all over Europe and Western Asia (Al-Snafi, 2019) and is used worldwide for its medicinal properties. For instance, in India, the leaves of *L. officinale* are used as a sedative and the whole aerial part for its diuretic, anti-gout, antipyretic and anti-inflammatory properties (Al-Snafi, 2019), while the roots are used as a decoction for eruptive diseases (Khare, 2007). Like *Echium* spp. or *Alkanna* spp., *L. officinale* is a valuable source of bioactive metabolites such as chlorogenic acid, rosmarinic acid, lithospermic acid and alkannin/shikonin and their derivatives (A/Sd); (Rai et al., 2018).

Alkannin/shikonin (A/S) are enantiomeric naphthoquinones produced in root tissues (Rai et al., 2018). They are natural lipophilic red pigments sequestered in the phospholipid layer and their accumulation in the apoplastic space are responsible for the red or purple colouration of roots (Brigham et al., 1999; Tatsumi et al., 2016). They are strongly involved in the antimicrobial activity of the plants that produce them and form a chemical barrier against soil-borne microorganisms (Brigham et al., 1999). Antibacterial (Papageorgiou et al., 1979), antifungal (Sasaki et al., 2002), as well as antiviral (Li et al., 2012) activities have been attributed to A/Sd. Moreover, A/Sd are also known for wound healing, regenerative, anti-cancer and anti-inflammatory properties (Papageorgiou et al., 2008).

Several factors can influence the biosynthesis of A/S. For instance, the presence of phytohormones such as methyl jasmonate (MeJA), ethylene, auxins and fungal elicitors are known to increase the production of A/Sd *in planta*. Whereas light, ammonium, or benzoic acid inhibit their production (Brigham et al., 1999; Pal et al., 2019). Recent studies were performed to elucidate the shikonin production pathway and its regulation *in planta*. For example, Skoneczny et al. (2019) used a metabolomic approach, profiling naphthoquinones and pyrrolizidine alkaloids and demonstrated the influence of high temperature and water withholding on the accumulation of A/Sd, especially deoxyshikonin, in *Echium plantagineum*. Similarly, shikonin derivatives were shown to accumulate over time in *Echium* spp. with higher amount in mature plants, showing an interesting pattern between perennial species such as *E. vulgare* and annual species such as *E. plantagineum* (Skoneczny et al., 2017). Also, Rai et al. (2018) performed a multi-omics analysis to propose 15 candidate genes involved in shikonin biosynthesis in *L. officinale*, and Fu et al. (2021) produced hairy roots of *E. plantagineum* to determine the role of a geranylhydroquinone 3″-hydrolase in shikonin production.

Despite *L. officinale* is a well-known species that produce A/Sd, its cultivation remains difficult and is particularly hindered by poor germination and lack of vegetative material (Yazaki, 2017). Moreover, this species needs a minimum of 2 years of development to reach acceptable contents of A/S for economically viable exploitation (Khosravi et al., 2018). To overcome these challenges, several studies have focused on increasing A/Sd production through chemical synthesis or plant tissue culture technologies. For example, *in vitro* cell cultures (Khosravi et al., 2018) have been developed for *L. officinale*. Cell cultures in suspension have the advantage of growing faster, and metabolites produced can be extracted more easily than from intact plants (Mulabagal and Tsay, 2004; Mustafa et al., 2011; Chandran et al., 2020). However, secondary metabolites are usually produced by specialised cells and some of them might not be produced in undifferentiated cell suspensions. These undifferentiated cultures render these systems also limited to study the biosynthesis of plant metabolites. Moreover, studying the effect of biotic factors on the plant metabolism is challenging under most tissue culture approaches as it requires reconciling the growth of the plant cell/tissue culture and another organism. Such *in vitro* systems deviate a lot from the natural conditions, and whole plant culture systems are thus preferable for understanding the metabolic interactions of the plant with its environment.

In natural habitats, plants interact with a wide range of microorganisms that can promote growth and the production of SMs in medicinal plants (Pang et al., 2021). The induction of plant SMs by bacteria can be linked to the modulation of plant defences (Erb and Kliebenstein, 2020; Stassen et al., 2021). Indeed, plants can recognise bacterial elicitors or microbe-associated molecular patterns (MAMPs), inducing defence responses. The defence responses can be systemic or local, involving signalling pathways such as the jasmonic acid (JA), salicylic acid (SA), ethylene (ET), abscisic acid (ABA), or the stimulation of reactive oxygen species (ROS) production (Zhang et al., 2018). The local response is expressed by oxidative bursts, cell wall composition changes, synthesis of phytoalexins and production of pathogenesis-related proteins (Ahuja et al., 2012; Isah, 2019). For example, Yang et al. (2019) showed that *Pseudomonas fluorescens* ALEB7B stimulated the production of sesquiterpenoids in the medicinal plant *Atractylodes macrocephala* by modulating the gibberellic acid and JA pathways. Therefore, using bacteria to improve SMs biosynthesis in medicinal plants might represent a suitable approach for increasing the production of therapeutical chemicals.

Despite the interesting properties of naphthoquinones and especially A/Sd (Mone et al., 2021), no studies have been performed so far on the induction of plant naphthoquinones by bacteria. In the present study, the objective was to assess the effects of bacteria on the production of A/Sd in *L. officinale*. To achieve this, *L. officinale* plants were grown under *in vitro* and greenhouse conditions in association with selected bacteria isolated from the roots of a closely related species, *Alkanna tinctoria* (Rat et al., 2021). These bacteria were selected based on preliminary results of A/Sd induction in hairy roots of *A. tinctoria* as shown in our previous study (Rat et al., 2021). Additionally, the selection was expanded to include bacterial groups that are often isolated from plants. The effects of the bacterial strains on the SMs production were evaluated through targeted and non-targeted metabolomics and supported by gene expression analysis.

## Materials and methods

### Plant culture and propagation

Clonal plant material of *L. officinale* L. was used for the experiments. Seeds (Rühlemann’s Kräuter & Duftpflanzen, Germany) were surface-disinfected by immersion in 100 ml of 5% sodium hypochlorite solution containing one drop (i.e., 5 μl) of tween 20 (Sigma-Aldrich, Germany) for 5 min and rinsed three times with sterile distilled water, adapted from Jafari et al. (2016). The sterilised seeds were germinated in plastic pots (1 l) containing a mixture of turf and perlite 2:1 (v:v) sterilised twice at 145°C in an oven during 10 h. The pots were then placed in a polytunnel greenhouse and watered on demand.

After 5 months of growth, each plant was used to produce a clonal lineage. To do so, every axillary node was cut (0.5–1 cm) and surface sterilised as follows: the explants were rinsed for 20 min under cold tap-water in a sieve with an aperture size of 250 μm, followed by a washing cycle with agitation in 70% ethanol for 20 s. They were then transferred to a 0.25% HgCl_2_ solution and gently stirred for 10 min. The explants were rinsed three times in sterile tap water and transferred to sterile 580 ml glass jars (Weck, Germany) containing 100 ml of Murashige and Skoog (Murashige and Skoog, 1962) medium (Duchefa Biochemie B.V., Netherlands). Jars were closed with a glass lid and cotton ring, allowing gas exchange. In the end, only one single lineage was kept so that each plant was rigorously similar.

The clones were finally grown routinely on a Murashige Skoog medium slightly modified (MS^mod^). To induce plant rooting, a different modification of MS medium (MS^mod rooting^) was adapted from the protocol of Gerardi (Gerardi et al., 1998). The chemical compositions of both media are presented in Supplementary Table S1.

### *In vitro* pre*-*screening of bacteria for the induction of A/Sd production by *Lithospermum officinale*

**Figure 1:**
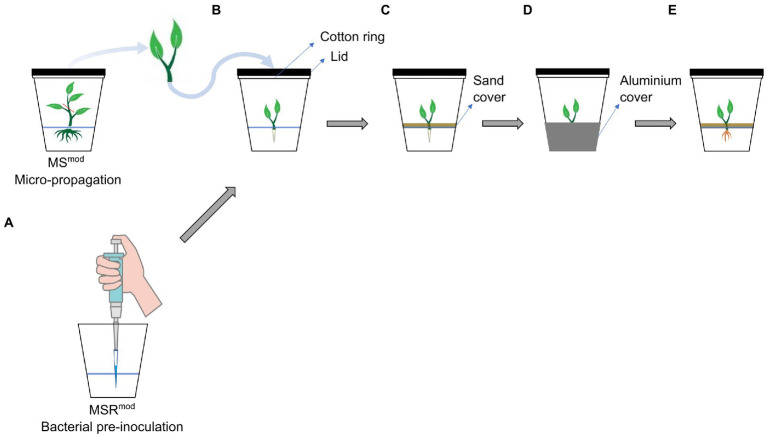
Schematic representation of the set-up for the *in vitro* screening of bacteria. **(A)** Medium inoculation with a bacterium prior to the transfer of the plants. **(B)** Transfer of the plants to the glass jar covered by a glass lid and a sterile cotton ring for gas exchange. **(C)** To avoid light in the root compartment, sterile sand was layered on the surface of the medium; **(D)** The bottom of the glass jar was covered with aluminium foil. **(E)** Root growth and pigmentation were observed 7 weeks later.

A whole-plant *in vitro* culture system was designed to grow *L. officinale* and to study the production of A/Sd following bacterial inoculation (Figure 1). A modified version of the Strullu-Romand medium (MSR) was used and named MSR^mod^. The MSR^mod^ medium contained gellan (Gelrite, Duchefa Biochemie B.V., Netherlands) as gelling agent, did not contain ammonia, as it is a known inhibitor of shikonin production (Fujita et al., 1981), nor sugar, since the plant was intended to be autotrophic (Supplementary Table S1).

A set of 50 bacteria previously isolated from another A/Sd-producing plant, *Alkanna tinctoria* (L.) Tausch, was selected based on diversity and on phenotypic traits tested *in vitro* (Rat et al., 2021). The 50 strains were first grown in 35 ml Reasoner’s 2A broth (R2B, Acumedia) at 28°C, 100 rpm for 72 h. Before preparing the inocula, 5 ml of each bacterial culture was sampled and used to estimate bacterial concentrations *via* counting of colony-forming units (CFU), while the remaining culture was used as inoculum. The inoculum was centrifuged at 4°C, 14000 rpm for 10 min, the supernatant was discarded, and the pellet was preserved in 2 ml of R2B medium supplemented with 10% glycerol. Bacterial pellets were then stored at −20°C until inoculation.

To prepare the inoculum, a pellet was suspended in 28 ml of sterile phosphate-buffered saline (PBS) at pH 7.4. The resuspended inoculum was then adjusted to a concentration of 10^4^–10^6^ CFU/ml, and 10 μl were used to inoculate each plant.

The bacterial suspension was first injected in the MSR^mod^ medium with a micropipette. Then, a shoot tip of *L. officinale* of 3.5 cm length was selected and the top two-three leaves were removed by cutting. The plant was finally transferred in sterile conditions and inserted in the medium at the positions where the bacteria had been injected. Plants treated with only PBS were used as non-inoculated controls. To evaluate root pigmentation induced by bacterial treatments, a positive control, used as pigmented root reference, was also included in the screening, consisting of MSR^mod^ supplemented with 10 μm MeJA, a strong elicitor of shikonin production (Yazaki et al., 1997; Yazaki, 2017). Two glass jars, each containing three plants were prepared per treatment. To avoid light in the root compartment, which can inhibit the production of shikonin (Yazaki, 2017), the surface of the medium was covered with sterilised (in an oven at 145°C during 10 h; adapted from Alkadhim, 2018) quartz sand and the lower half of the jar was wrapped with aluminium foil (Figure 1). The jars were then incubated in a growth chamber at 20°C, 16:8 h light: dark, with a light intensity of 50 μmol m^−2^ s^−1^. Plants were harvested for analysis after 7 weeks of incubation, and the effect of each bacterial treatment on root architecture and pigmentation was assessed visually as an indication of A/Sd production.

### Induction of production of A/Sd by selected bacteria under *in vitro* and greenhouse conditions

A similar *in vitro* experiment as mentioned previously was conducted on a selection of the most effective bacteria from the pre-screening. Six glass jars with three plants each were used per treatment. Each jar containing three plants was thus considered as a biological replicate; six biological replicates per treatment were used. The treatments consisted of inoculating bacteria as described above. PBS was used as a negative control treatment. Plants were grown for 7 weeks. The root system of each plant was harvested separately, cleaned with distilled water, gently dried with tissues and fresh weight was recorded before lyophilisation. The dry weight was then measured, and the samples stored at −80°C for analysis of the content of A/Sd by targeted and non-targeted metabolomics (see metabolomic analysis section).

A greenhouse experiment was also conducted in parallel with the same bacteria. Three-week-old rooted, *in vitro* produced, plants were used (Supplementary Figure 1). The experimental design consisted of seven treatments (six bacteria and one control), with 10 biological replicates (1 plant/pot) for every treatment. The plants were transferred to trays of 108 pots of 3 cm diameter filled with twice sterilised (in an oven at 145°C during 10 h) substrate, composed of calcinated clay and quartz sand of two size categories (0.4–0.8 mm and 1–2 mm) in a proportion of 2:2:1 (v:v:v). At the time of transfer to the trays, every plant was inoculated with 1 ml of the bacterial culture resuspended in PBS at a concentration of 10^6^ CFU/ml. The plants were extracted from the agar-medium and the roots were rinsed with sterilised tap water (autoclaved 15 min at 121°C). They were then aseptically placed in the pots. The bacteria were inoculated directly on the roots and covered with the substrate. The control treatment was inoculated with 1 ml of sterilised PBS. Each tray was sprayed with water and placed inside a plastic hood for acclimatisation in the greenhouse, under natural light and uncontrolled temperature conditions, with temperatures outside ranging from 20 to 33°C. After 4 weeks of acclimatisation, the full root-system of every plant, with the rhizosphere, was transferred to a 1 l pot containing the same substrate. In addition, a second inoculation treatment was performed, every plant was inoculated with 1 ml of the bacterial culture resuspended in PBS at a concentration of 10^6^ CFU/ml as follows: using a micropipette, every bacterial treatment was injected in the substrate, besides the stem, at the point where the main root starts growing. The positions of the pots in the greenhouse were randomised (Supplementary Figure 1), and plants were watered three times a week, two times with tap water and one time with 100 ml of Hoagland solution (Hoagland and Arnon, 1950). The plants were then grown for 2 months.

After this period, shoot length and fresh weight of the shoot and roots of each biological replicate were recorded. For the shoots, dry weight was measured after drying at 70°C for 72 h. The root system of each plant was lyophilised. The dry weight was recorded, and samples further stored at −80°C for analysis of the content of A/Sd by HPLC-DAD.

### Metabolomic analysis

The lyophilised roots were ground to fine powder using a ball mill (Fritsch Pulverisette 0, Germany). For the *in vitro* tests, root systems of each jar (3 plants/jar) were pooled to have enough biomass. For the greenhouse test, no pooling was required and individual root systems (e.g., one replicate) were used for analysis. For each powdered sample, a subsample of 35 mg was placed into microcentrifuge tubes for SMs extraction with 1.5 ml of methanol (LC–MS grade, Honeywell Riedel de Haën, United States) in an ultrasound bath at 10% power for 3 h (Bandelin Sonorex Digital 10P, Berlin, Germany) followed by centrifugation for 10 min at 12500 rpm (Hermle Z 216 MK, Wehingen, Germany). The supernatants were collected and subjected to targeted analysis by High-Performance Liquid Chromatography-Diode Array Detection (HPLC-DAD) and non-targeted Ultra-High-Performance Liquid Chromatography-High Resolution Mass Spectrometry (UHPLC-HRMS) analysis, after filtering with 0.22 μm polytetrafluorethylene (PTFE) filters.

In order to identify each derivative of A/S for further quantification by HPLC-DAD, calibration was performed with fully identified A/S standards in methanol (Supplementary Figure 2): alkannin (Ikeda, Japan), shikonin (Ichimaru, Japan), acetylshikonin (ABCR GmbH, Germany), propionylshikonin (synthesised by Prof. Elias Kouladouros, Agricultural University of Athens, Greece, purity identification by A. Assimopoulou), deoxyshikonin (TCI, Belgium), β,β–dimethylacrylshikonin (ABCR GmbH, Germany) and isovalerylshikonin (TCI, Belgium). Acetonitrile (HPLC-grade, Honeywell Riedel de Haën, United States), ultrapure water (Merck Millipore, Germany) and formic acid (HPLC-grade, Merck KGaA, Germany) were used to perform the HPLC analysis. For UHPLC-HRMS analysis, formic acid (LC–MS reagent grade, Honeywell Fluka, United States) was added to both water and methanol (LC–MS grade, Honeywell Riedel de Haën, United States; 0.1% v:v).

Targeted analysis by HPLC with DAD detection set at 520 nm wavelength was chosen for the quantitation of A/Sd. Analyses were performed on an ECOM analytical HPLC instrument, model ECS05 (Prague, Czech Republic), using a Fortis SpeedCore C18 column (Cheshire, United Kingdom). The mobile phase comprised ultrapure water (A) and acetonitrile (B). Each run lasted 13 min with a flow rate of 1 ml/min, and samples were run in a randomised sequence to avoid bias, example chromatograms in Supplementary Figure 2. Data were processed with the software Clarity (DataApex, Prague, Czech Republic). Elution was performed using the following solvent gradient: 0 min 30A/70B, 8 min 100B, 13 min 100B. Prior to the next injection, the column was equilibrated for 5 min with the initial solvent composition. The column temperature was kept at 35°C.

Data for non-targeted metabolomics by UHPLC-HRMS on root extracts from plants grown *in vitro* were recorded on a Q Exactive™ Focus hybrid quadrupole-Orbitrap MS (Thermo Scientific, Waltham, Massachusetts, United States) instrument. The column used was an Acquity UPLC HSS C18 SB 1.8 μm 2.1 × 100 mm (Waters, Milford, Massachusetts, United States), thermostated at 50°C, with the mobile phase flow rate set to 0.3 ml/min. The solvents used were ultrapure water (A) and methanol (B), both with 0.1% (v:v) formic acid. The gradient elution program was as follows: 0 min 95A/5B, 1 min 50A/50B, 8 min 0A/100B, 13 min 0A/100B, 13.01 min 95A/5B, 16 min 95A/5B. Data were acquired in positive ionisation mode, with the capillary temperature set to 320°C using the instrument’s MS/MS discovery feature. The normalised collision energy was set to 35 eV. The instrument control, acquisition and initial processing of the data were conducted by the Xcalibur software (version 4.1, Thermo Scientific, USA). Furthermore, data alignment and feature extraction were performed on the XCMS online platform (Huan et al., 2017). Identification of detected compounds was performed using the software Compound Discoverer (version 3.2, Thermo Scientific, USA).

A hypothetical metabolic network was then elaborated based on the compounds detected and identified by UHPLC-HRMS, according to the description of individual pathways as previously reported (Brown, 1962; Pollier and Goossens, 2012; Rai et al., 2018; Torrens-Spence et al., 2019; Kianersi et al., 2020). Information from the KEGG database was also compared for validating the proposed metabolic network (Ogata et al., 1999).

### qPCR assay to assess the effect of bacteria on the expression of the gene *PGT* involved in the production of A/Sd

An independent inoculation experiment was set up to evaluate the effect of an A/Sd-inducing strain (*Chitinophaga* sp. R-73072) and a non-inducing strain (*Rhizobium* sp. R-72433) on the expression of the *p*-hydroxybenzoate geranyltransferase gene *PGT* in *L. officinale* roots by qPCR. The gene *PGT* codes for LePGT1, an enzyme catalysing the reaction of geranylpyrophosphate with *p*-hydroxybenzoic acid to form geranylhydroxybenzoic acid, a direct precursor of A/S (Tang et al., 2019). Briefly, three treatments representing an A/Sd-inducing strain, a non-inducing strain and a PBS control were included in the *in vitro* cultivation system as previously described. Each treatment contained three biological replicates and plants were grown for 4 weeks.

Plants were harvested and roots frozen in liquid nitrogen and homogenised with a MM400 Mixer Mill (Retsch, France) for 2 min at 25 Hz. The total RNA was extracted using the RNeasy Plant Mini kit (Qiagen, Germany). The extraction was performed according to the manufacturer’s instructions using RLT buffer and in-column DNAse treatment. A Bioanalyzer PicoChip (Agilent technologies, United States) was used to quantify RNA samples and a Nanodrop 2000 was used to measure the purity. Next, cDNA was produced using the SuperScript IV VILO Master Mix with ezDNase treatment (Invitrogen, United States) and qPCR analysis was performed with the PowerTrack SYBR Green Master Mix (Applied Biosystems, United States) in a Lightcycler 480 instrument (Roche, Switzerland), according to manufacturer’s instructions. Primer sequences are listed in Supplementary Table S2.

### Statistical analysis

The data obtained from HPLC-DAD were used to compare the effect of the six bacterial inoculants used in the *in vitro* and greenhouse experiments on the total A/Sd content (mg per kg of root, sum of all A/Sd detected) and on plant dry weight (g). For the *in vitro* data, the statistical analysis was performed on six biological replicates per treatment. For the greenhouse data, the statistics were performed on ten biological replicates per treatment. The normal distribution of the residuals was verified with the Shapiro–Wilk normality test. Then, the appropriate comparison test was performed.

## Results

### *In vitro* pre-screening of bacteria for the induction of A/Sd production by *Lithospermum officinale*

Fifty strains were screened for A/Sd induction and visual inspection of root pigmentation and development on inoculated plants revealed three distinct bacterial groups, in comparison with the negative and positive controls. Group one consisted of 32 strains that did not induce root pigmentation nor hinder root growth. Group two was defined by 13 strains that induced pigmentation but did not impair root growth. Finally, five strains that induced root pigmentation and inhibited root growth were classified in group three (Supplementary Table S3).

**Figure 2:**
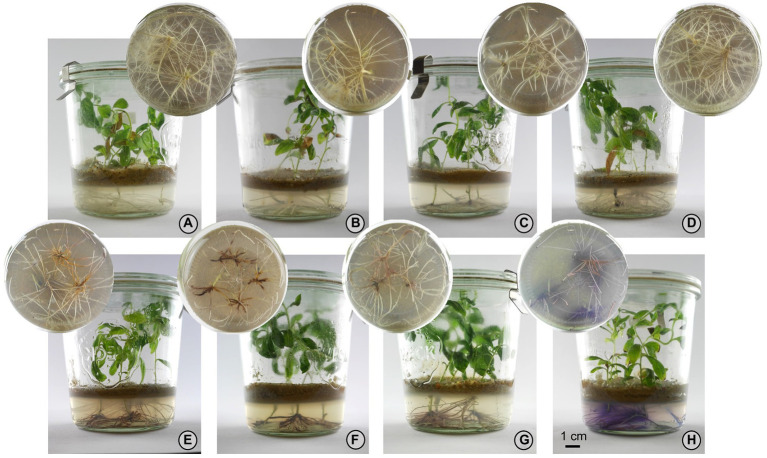
Effect of different bacteria on root development and A/Sd production by *Lithospermum officinale*. **(A)** Negative control (non-inoculated), **(B)** *Rhizobium* sp. R-72433, **(C)** *Chitinophaga* sp. R-72269, **(D)** *Brevibacterium* sp. R-71875, **(E)** *Pseudomonas* sp. R-71838, **(F)** *Xanthomonas* sp. R-73098, **(G)** *Chitinophaga* sp. R-73072, **(H)** Positive control (MSR^mod^ + MeJA 10 μm). Treatments shown in panels **(A,B)** do not induce root colouration; treatments shown in panels **(C–G)** were confirmed to induce visible colouration of roots and adjacent medium due to production of A/Sd. Strong reaction in the positive control **(H)** is accompanied by exudation in the medium. From **(A–H)**, we can also observe a decrease in root branching, from normal root growth in the control treatment **(A)**, to less branching and secondary roots in panel **(H)**.

Among the 13 bacterial strains from group two, five were selected to confirm their effect on A/Sd induction under *in vitro* and greenhouse conditions: *Pseudomonas* sp. R-71838, *Chitinophaga* sp. R-73072, *Xanthomonas* sp. R-73098, *Brevibacterium* sp. R-71875, *Chitinophaga* sp. R-72269. Furthermore, *Rhizobium* sp. strain R-72433, a representative from group one, was also tested as a non-A/Sd-inducing treatment (Figure 2).

### Induction of A/Sd production by selected bacteria under *in vitro* and greenhouse conditions

**Figure 3:**
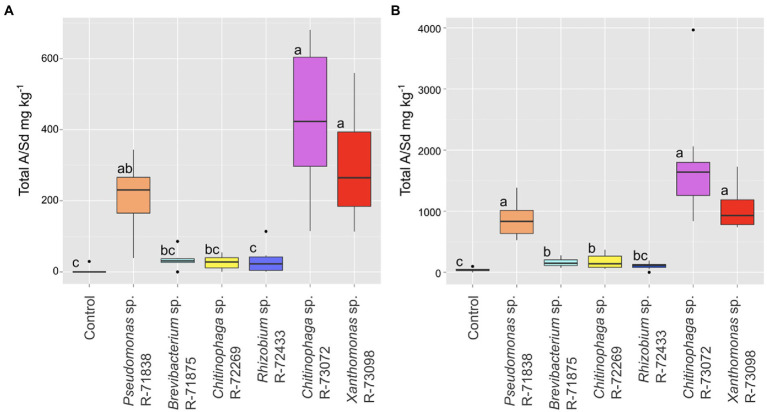
Boxplot representations of the median (thick black line), upper and lower quartiles (box), for the total A/Sd content in the root of *L. officinale* inoculated with selected bacteria. **(A)** Results under *in vitro* conditions (6 biological replicates per treatment). **(B)** Results under greenhouse conditions (10 biological replicates per treatment). Data were analysed by a Kruskal–Wallis test followed by a Dunn test (*p* < 0.05). Treatments associated with the same letter did not differ significantly.

To confirm their effect on the production of A/Sd, the six strains selected above were inoculated *in vitro* on plants of *L. officinale.* At the end of the growth period, roots were harvested, and HPLC-DAD was used to quantify total A/Sd content. The residuals did not follow a normal distribution. A Kruskal–Wallis test conducted on the data showed that some bacterial treatments significantly impacted the total A/Sd (value of *p* of 2.05e^−05^). According to the Dunn test (Figure 3A), the strains *Chitinophaga* sp. R-73072, *Xanthomonas* sp. R-73098 and *Pseudomonas* sp. R-71838 induced a significantly higher A/Sd production *in planta* than the non-inoculated control. *Chitinophaga* sp. R-73072, *Xanthomonas* sp. R-73098 also induced a significantly higher A/S production than the strains *Brevibacterium* sp. R-71875, *Chitinophaga* sp. R-72269 and *Rhizobium* sp. R-72433 (Figure 3A). The strains R-71875 and R-72269 did induce pigmentation in the screening experiment but the total A/Sd content of the inoculated plants did not differ statistically from the non-inducing strain R-72433 and the non-inoculated control. They are thus not considered as A/Sd-inducers anymore.

In the greenhouse experiment, statistical analysis (Kruskal–Wallis) showed a similar trend to the *in vitro* experiment (value of *p* 5.70e^−11^). The best inducers of A/Sd were *Chitinophaga* sp. R-73072, *Xanthomonas* sp. R-73098 and *Pseudomonas* sp. R-71838 (Figure 3B). Again, the strains R-71875 and R-72269 did not induce more A/Sd than the non-inducing strain R-72433. The bacterial effect on A/Sd induction was reproducible between *in vitro* and greenhouse assays. However, the A/Sd content in greenhouse conditions was about fourfold higher than under *in vitro* conditions for all treatments.

The shoot length was also measured for each treatment. The residuals values on this data followed a normal distribution. The results showed that all the bacterial treatments significantly increased shoot height as compared to the non-inoculated control plants (Supplementary Table S4). Similarly, shoot dry weight was significantly higher for all bacterial treatments as compared to the non-inoculated control plants, except for *Xanthomonas* sp. R-73098 and *Rhizobium* sp. R-72433. Root dry weight data followed a normal distribution and, according to the one-factor ANOVA followed by Newman–Keuls test, the root dry weight was significantly higher for *Chitinophaga* sp. R-72269 treatment as compared to the non-inoculated control. Nonetheless, no significant difference was observed between the different bacterial treatments for plant growth (Supplementary Table S5).

### Non-targeted metabolomic analysis of *Lithospermum officinale* grown *in vitro* under different bacterial treatments

**Figure 4:**
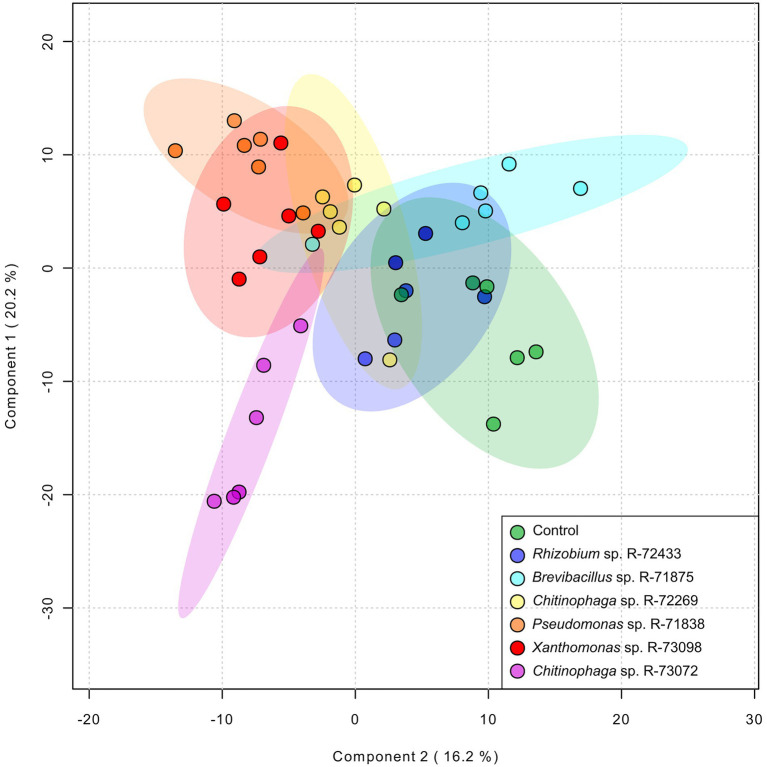
Partial least squares discrimination analysis (PLS-DA) of non-targeted metabolomics data to assess the impact of bacterial treatments on the metabolome of *L. officinale* plants grown *in vitro*. Plants were inoculated with *Chitinophaga* sp. R-73072, *Pseudomonas* sp. R-71838, *Xanthomonas* sp. R-73098, *Chitinophaga* sp. R-72269, *Rhizobium* sp. R-72433 and *Brevibacterium* sp. R-71875. Control plants were prepared by inoculating them with PBS. The PLS-DA diagram contains all identified and non-identified features from the non-targeted metabolome as processed by the online server MetaboAnalyst 5 (module chemometrics analysis). Axes 1 and 2 represent up to 36.4% of the data variation and the model was predicted with a cross-validation accuracy of 0.4523, *R*^2^ 0.3943 and *Q*^2^ 0.2827 for these two components.

Following the quantification of the total A/Sd content in the roots of *L. officinale* plants grown *in vitro* and inoculated with six different bacteria, a non-targeted metabolomic analysis was performed. A total of 9,624 features were detected by UHPLC-HRMS. Features detected within the different treatments were subjected to partial least squares discriminant analysis (PLS-DA; Figure 4). The discriminant analysis showed differences between individual bacterial treatments on the root metabolome. Treatments with *Rhizobium* sp. R-72433, *Brevibacterium* sp. R-71875 and *Chitinophaga* sp. R-72269 overlapped with the non-inoculated control. Therefore, those bacteria did not have a significant impact on the plant root’s metabolome. Conversely, *Pseudomonas* sp. R-71838, *Xanthomonas* sp. R-73098 and especially *Chitinophaga* sp. R-73072 induced remarkable metabolomic changes in the plants.

**Figure 5:**
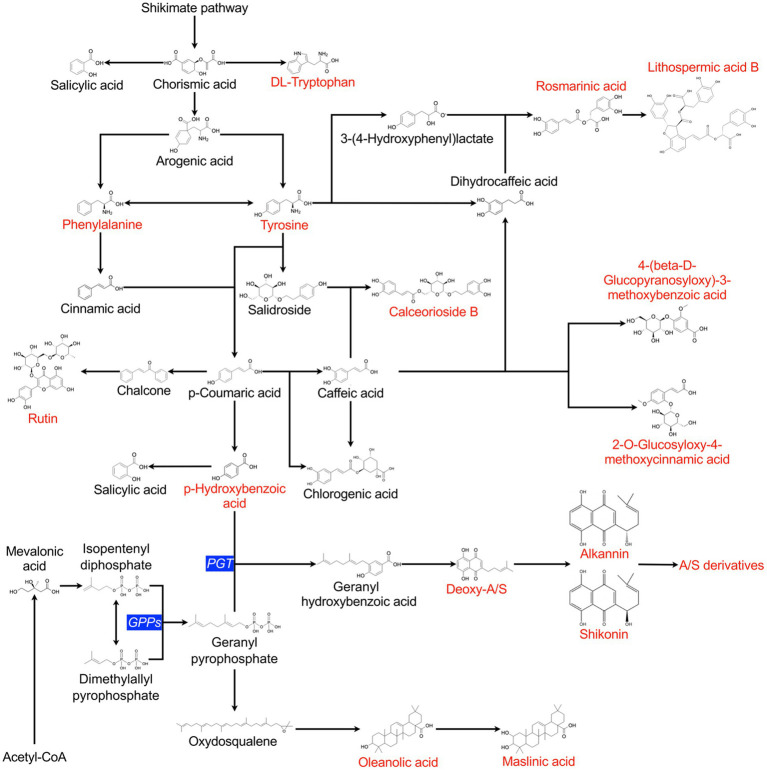
Schematic secondary metabolism pathways in *L. officinale* plants grown *in vitro*. Compounds depicted in red were detected and identified by UHPLC-HRMS. The metabolic network was built based on literature reports and data derived from the KEGG database. Critical enzymes for the synthesis of A/S are noted in blue. PGT, *p*-hydroxybenzoate geranyltransferase; *GPPs*, geranyl pyrophosphate synthase.

From the complete dataset of detected features, 25 compounds were identified (Supplementary Table S6). The nature of several of the identified compounds indicated that they were produced from the shikimate pathway *via* chorismic acid, illustrating that several branches of the shikimate pathway were highly active and responded to bacterial stimulation. The shikimate pathway is associated with SMs, especially phytoalexins or defensive compounds (Tzin and Galili, 2010). Figure 5 shows the proposed metabolic network correlating the different identified compounds. The correlated occurrence of compounds was analysed to elucidate the regulation of A/Sd and other SMs (Supplementary Figure 3). Correlations confirmed the links between the production of compounds and their common metabolic intermediates or immediate precursors. For example, oleanolic and maslinic acid were correlated positively, as well as rosmarinic acid and lithospermic acid B. Moreover, acetyl-A/S and deoxy-A/S were positively correlated with rosmarinic acid, and they were both correlated with *p*-hydroxybenzoic acid. These compounds share two common precursors, tyrosine and *p*-coumaric acid (Figure 5), which might explain the link between these two molecules. Besides, *p*-hydroxybenzoic acid also presented a positive correlation with oleanolic and maslinic acid, whereas the propionyl-A/S, was negatively correlated with oleanolic and maslinic acids (Supplementary Figure 3). Additionally, we observed positive correlations between lithospermic acid B, 2-O-glucosyloxy-4-methoxycinnamic acid and 4-(beta-D-glucopyranosyloxy)-3-methoxybenzoic acid. Conversely, a negative correlation was observed between the former compounds and calceorioside B.

**Figure 6:**
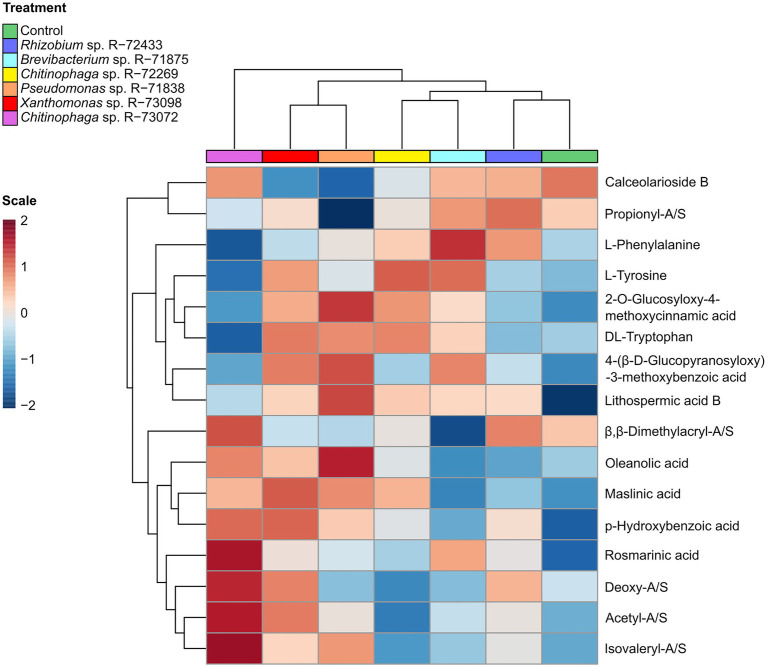
Correlations of identified plant metabolites clustered by treatment. Treatments include non-inoculated control and six bacterial treatments (6 biological replicates per treatment). Peak intensity data were analysed whit the online server MetaboAnalyst 5, module hierarchical clustering (heatmaps). Euclidean distance was used for clustering, considering average intensity values of every metabolite calculated per treatment (every treatment included six biological replicates). Metabolite intensity values were auto-scaled using standard settings (range from 2 to −2) for visual representation in the heatmap.

The effect of bacterial treatments on the metabolome of *L. officinale* is illustrated in Figure 6. In general, there was a noticeable difference between bacterial treatments and non-inoculated control. The non-inoculated control treatment presented a slightly higher production of calceorioside B and downregulation in the production of most of the other identified metabolites. The treatment with *Chitinophaga* sp. R-73072 involved the induction of compounds such as A/Sd and oleanolic/maslinic acids, whereas plants treated with the other *Chitinophaga* sp. R-72269 did not show such strong metabolic changes compared to the control.

*Pseudomonas* sp. R-71838 induced a much higher production of lithospermic acid B compared to the other treatments and it was also associated with higher production of 2-O-glucosyloxy-4-methoxycinnamic acid, 4-(beta-D-glucopyranosyloxy)-3-methoxybenzoic and downregulation in the production of calceorioside B.

When comparing the effect of the inducing treatments, *Xanthomonas* sp. R-73098 showed a general induction of many compounds of the specialised metabolome compared to *Pseudomonas* sp. R-71838 or *Chitinophaga* sp. R-73072.

Finally, the other treatments that induced only slight metabolic changes compared to the control were *Rhizobium* sp. R-72433 and *Brevibacterium* sp. R-71875. These treatments were positively correlated with L-phenylalanine and calceorioside B but did not show a strong correlation with other identified compounds involved in plant defence.

### Effect of bacteria on the expression of gene *PGT* involved A/Sd production

The A/Sd-inducing strain *Chitinophaga* sp. R-73072 and the non-inducing strain *Rhizobium* sp. R-72433 were tested for their effect on the expression of the plant gene *PGT*. Statistical comparison on the expression of *PGT* showed that both bacterial treatments influenced the expression level (value of p of 0.007), nevertheless *Chitinophaga* sp. R-73072 influenced slightly higher levels of induction compared to the control and non-inducing treatment. The detailed result of the ANOVA test is shown in Supplementary Table S7.

## Discussion

Secondary metabolites play a major role in the adaptation of plants to biotic and abiotic stresses. This adaptation is regulated by signalling pathways (i.e., JA, SA, ABA and ET) as well as ROS, which influences the accumulation of plant SMs (Zhang et al., 2018), including A/Sd (Yazaki et al., 1997), rosmarinic acid (Mizukami et al., 1993) and oleanolic acid (Vergara Martínez et al., 2018). Among the biotic factors, bacteria are known to influence plant signalling pathways and the production of plant SMs (Pang et al., 2021). This work aimed to elucidate the effect of bacterial strains on the production of A/Sd and other SMs in *L. officinale*.

To address this question, we developed a novel whole-plant *in vitro* cultivation system (Figure 1) offering numerous advantages over classical pot systems. These are the absence of unwanted microbial contaminants, the easy observation of root development and changes in pigmentation induced by bacterial inoculation, the reproducibility of results and their good correlation with those obtained in the greenhouse. Therefore, this whole plant *in vitro* culture system proved to be an efficient tool to study the effect of plant-microbe interactions on SMs production, especially A/Sd.

A selection of 50 bacterial strains were first tested *in vitro* for their effect on root architecture and pigmentation. Three different patterns of plant response were observed. Thirty-two bacteria did not inhibit root development but did not induce any pigmentation and were thus disregarded further. Five strains impaired root development and were thus disregarded, despite their impact on the production of red pigments in the roots. As we inoculated small plants without roots, the induction of ROS and the concomitant oxidative burst influenced by the bacteria may have led to these observations. Finally, thirteen bacterial strains did not inhibit root growth and induced pigmentation.

Of the six bacteria selected in the screening test, three (*Chitinophaga* sp. R-73072, *Xanthomonas* sp. R-73098 and *Pseudomonas* sp. R-71838) induced a significantly higher content of total A/Sd in the roots as compared to the non-inoculated controls, both *in vitro* and in the greenhouse. *Brevibacterium* sp. R-71875 and *Chitinophaga* sp. R-72269, initially selected for their pigmentation-inducing effect, did not induce more A/Sd than *Rhizobium* sp. R-72433 or the non-inoculated control and were thus considered finally as non-A/Sd inducing strains. The accumulation of other phenolic compounds rather than A/Sd could likely have caused such effect.

The induction of A/Sd was about fourth-fold higher in plants grown under greenhouse conditions than in plants grown *in vitro*. Such results confirmed previous observations on the comparison of SMs production in plants grown *in vitro* and under greenhouse conditions (Al-Qudah et al., 2011). In our study, the plants under greenhouse conditions were grown for a longer time (about 11 weeks) and at higher and more variable temperatures (uncontrolled in the greenhouse, outside temperature 20–33°C) compared to the *in vitro* system (7 weeks, 20°C), which likely enhanced the production and accumulation of A/Sd compared to the *in vitro* cultivation system. Moreover, in contrast to the *in vitro* cultivation system, plants were more exposed to variations in light intensity, temperature and humidity under greenhouse conditions. These factors have been reported to influence plant SM production. For example, Jansen et al. (2009) have shown that higher temperatures resulted in higher alkaloid content in *Lupinus angustifolius* seeds.

A/Sd have antimicrobial properties (Brigham et al., 1999; Rat et al., 2021) and can thus be considered as plant defensive compounds. It is well known from the literature that MAMPs activate the plant defense system, resulting in SM accumulation (Ahuja et al., 2012; Isah, 2019). In the study by Rat et al. (2021), *Chitinophaga* sp. R-73072, *Pseudomonas* sp. R-71838 and *Xanthomonas* sp. R-73098 were shown to express pectinase or ligninase activities (*in vitro*), whereas *Chitinophaga* sp. R-72269 did not show the ability to degrade pectin, lignin, or cellulose. Production of such enzymes for nutrient recycling or plant colonisation might induce plant defence response, resulting in the biosynthesis of plant antimicrobials (Pinski et al., 2019). Bacterial mutants deficient in hydrolytic enzymes production could be made and inoculated to plants to verify this hypothesis.

To gain insights into the induction mechanisms of A/Sd, a non-targeted metabolomics analysis was performed. Positive correlations were observed between certain compounds. For example, the correlation between A/Sd and rosmarinic acid was already observed in the study by Yamamoto et al. (2000). Moreover, we showed that *p*-hydroxybenzoic was positively correlated with rosmarinic acid and A/Sd, as well as with oleanolic and maslinic acids. Like A/Sd, rosmarinic acid, oleanolic and maslinic acids are phytoalexin-like plant defence compounds and the co-occurrence of *p*-hydroxybenzoic and several phytoalexin-like compounds might be linked with a general activation of the plant defence system. Notably, another A/Sd, propionyl-A/S, was negatively correlated with oleanolic and maslinic acids (Supplementary Figure 3). Oleanolic acid is derived from farnesyl pyrophosphate (FPP), and A/Sd compounds have geranylpyrophosphate (GPP) as one of their precursors. GPP is formed from isopentenyl pyrophosphate (IPP) and dimethylallyl pyrophosphate (DMAPP) while FPP is formed by the reaction of IPP with DMAPP or GPP. Oleanolic acid and A/Sd thus share GPP as a common precursor. In *L. erythrorhizon*, a recombinant FPP synthase (FPPs) homologue was shown to produce GPP from DMAPP and IPP but was unable to lead to the production of FPP and was thus described as a new kind of GPP synthase (*GPPs*). This *GPPs* is cytosolic and was correlated with the accumulation of A/S in *L. erythrorhizon* cells (Ueoka et al., 2020). The specific activity of this *GPPs* and the negative correlation observed in our study between propionyl-A/S and oleanolic acid may suggest that, under induction, the enzymatic reaction involving geranylpyrophosphate is driven in favour of A/Sd production and not of oleanolic acid production. Furthermore, we observed negative correlations between lithospermic acid B and calceorioside B. In literature, it has been shown that calceorioside B and lithospermic acid B share tyrosine and caffeic acid as precursors (Figure 5). Our data suggest that the production of these two compounds may involve competition for common precursors. Taken together, correlations between compounds in our metabolic data support the previously established connections illustrated in the metabolic pathways (Figure 5).

Besides the correlations between identified compounds, the effect of bacterial treatments on the *L. officinale* root metabolome was investigated. Non-targeted metabolomics showed that the non-inoculated control treatment was positively correlated with calceorioside B and negatively correlated with most of the other identified metabolites. As previously described, the production of metabolites, like A/Sd, lithospermic acid B and maslinic acid is related to the activation of the plant defence system. Our results suggested that the high content of calceorioside B may be linked with a basal state of the plant in which the defence system is not activated.

The treatment with *Chitinophaga* sp. R-73072 involved the induction of compounds such as A/Sd and oleanolic/maslinic acids. This highlighted the ability of the bacteria to stimulate the production of specific metabolites such as A/Sd, with oleanolic/maslinic acids likely side products of *GPPs*. Conversely to *Chitinophaga* sp. R-73072, the other *Chitinophaga* sp. strain, R-72269, did not induce great changes in the root metabolome compared to the control. This observation suggested that the response of the plant when challenged by *Chitinophaga* sp. R-73072 is specific for the induction of A/Sd. Among the A/Sd that were upregulated by R-73072, we could highlight the presence of derivatives like acetylshikonin and β,β–dimethylacrylshikonin. These two shikonin derivatives were described by previous research as promising drugs for therapeutic and biomedical applications related to the treatment of different cancer cell lines (Chen et al., 2002; Boulos et al., 2019; Guo et al., 2019). Our results, therefore, might find an application to secure the production of valuable molecules of medical interest.

Interestingly, Fazal et al. (2021) demonstrated the ability of two other shikonin producers, *Echium plantagineum* and *Lithospermum erythrorhizon,* to recruit bacteria from the genus *Chitinophaga* as part of their microbiomes. Here they also correlated the colonisation of different bacterial groups and the production of the plant’s secondary metabolites. These findings are in agreement with our results that suggest the ability of certain *Chitinophaga* spp. to colonise and induce A/Sd in different Boraginaceae plant species.

Inoculation with *Pseudomonas* sp. R-71838 was associated with a higher production of lithospermic acid B compared to the other treatments. Nevertheless, there was still production of A/Sd, as demonstrated by the HPLC results. As lithospermic acid B and A/Sd share a metabolic precursor (*p*-coumaric acid), it is possible that a strong induction of lithospermic acid B led to the side production of A/S compounds. In previous studies using tissue cultures of *L. erythrorhizon* with chemical stimulation, an equal production of lithospermic acid B and A/Sd was observed (Yazaki, 2017). Our work in *L. officinale* showed that these compounds seem likely to be negatively correlated (Supplementary Figure 3), illustrating the importance of using whole plant culture systems for studying the A/S pathway.

The effect of *Xanthomonas* sp. R-73098 was less targeted towards the induction of particular metabolites but instead activated the plant’s general metabolic response.

Treatments with non-A/S inducing strains *Brevibacterium* sp. R-71875 and *Rhizobium* sp. R-72433 were positively correlated with L-phenylalanine and calceorioside B induction but did not show a strong correlation with A/Sd or other identified compounds involved in plant defence (Figure 6).

To further elucidate the effect of bacteria on the A/S production, the expression level of the gene *PGT* (family *p*-hydroxybenzoate geranyltransferase), encoding LePGT1, known as a key enzyme of the A/S pathway (Tang et al., 2019), was also evaluated. Compared to the non-inoculated control, *PGT* was overexpressed in both the A/Sd-inducing and non-inducing bacterial treatment. However, our metabolomics data showed that only *Chitinophaga* sp. R-73072 and not *Rhizobium* sp. R-72433, increased A/Sd production.

It has been demonstrated that another gene coding for a geranylhydroquinone-3″-hydroxylase is also essential to produce A/S compounds. This geranylhydroquinone-3″-hydroxylase leads to the formation of 3″-hydroxy-geranylhydroquinone from geranylhydroquinone, which is then converted either to shikonin compounds or to shikonofuran compounds (Tang et al., 2019). Moreover, in a study conducted on *Arnebia euchroma* cells, Wang et al. (2014) demonstrated that the application of MeJA on shikonin-deficient cell lines led to the overexpression of several genes involved in the biosynthesis of A/Sd, including *PGT*, without resulting in the actual production of A/Sd. These results demonstrated that the *PGT* gene alone is not enough to assure the production of these compounds. The production of A/Sd seems to likely result from the co-expression of several genes. Our results with *Chitinophaga* sp. R-73072 and *Rhizobium* sp. R-72433 that both induced *PGT* but resulted in different plant phenotypes, seem to confirm this hypothesis. Therefore, we can suggest that the interaction of the plant with either R-72433 or R-73072 may lead to a differential expression of several genes involved in the A/S pathway and these strains may be used further to unravel the elusive metabolic processes occurring after the PGT-catalysed reaction.

In this work, we designed and tested a whole plant *in vitro* cultivation system that allowed effective screening of bacteria for inducing the production of A/Sd *in planta*, opening new perspectives towards the application of microorganisms to improve the production of valuable phytochemicals in plants for industrial exploitation. Besides A/Sd, oleanolic and maslinic acids as well as calceorioside B were detected for the first time in *L. officinale*, and new correlations between compounds were established. Our results revealed specific regulation patterns in the production of secondary metabolites in *L. officinale* and pave the road for further studies on this plant with interesting perspectives for industry and pharma.

## Data availability statement

The datasets presented in this study can be found in online repositories. The names of the repository/repositories and accession number(s) can be found at: This data is available at the NIH Common Fund's National Metabolomics Data Repository (NMDR) website, the Metabolomics Workbench, https://www.metabolomicsworkbench.org, where it has been assigned Project ID PR001283. The data can be accessed directly via it's Project doi: 10.21228/M8VX1B.

## Author contributions

AVA, HN, and AR designed and performed the research, analysed the data, and wrote the manuscript. NR performed the research and data acquisition, analysed the data, and wrote the manuscript. CIN contributed to UPLC-MS run and data acquisition. DAL contributed to resources (UPLC-MS instrument) and data acquisition. ANA, SD, and CS critically reviewed and edited the manuscript and contributed to funding acquisition. PR designed the research and critically reviewed and edited the manuscript. AW designed the research, critically reviewed and edited the manuscript, and contributed to funding acquisition. All authors contributed to the article and approved the submitted version.

## Funding

This research was supported by the European Union’s Horizon 2020 research and innovation program under the Marie Skłodowska-Curie grant agreement No. 721635, MICROMETABOLITE ITN project (http://micrometabolite.eu/), from the Special Research Fund BOF of Ghent University (Grants 01IT0720 and 01IT0121) and NIH grant, U2C-DK119886.

## Conflict of interest

AVA, PR, and CS were employed by Institut für Pflanzenkultur GmbH & Co. KG.

The remaining authors declare that the research was conducted in the absence of any commercial or financial relationships that could be construed as a potential conflict of interest.

## Publisher’s note

All claims expressed in this article are solely those of the authors and do not necessarily represent those of their affiliated organizations, or those of the publisher, the editors and the reviewers. Any product that may be evaluated in this article, or claim that may be made by its manufacturer, is not guaranteed or endorsed by the publisher.
