# Supplementary material for: Root-associated bacteria modulate the specialised metabolome of Lithospermum officinale L
Source: Front Plant Sci. 2022 Aug 30;13:908669. doi: 10.3389/fpls.2022.908669 (PMC9468582; doi:10.3389/fpls.2022.908669)
Supplement: Supplementary file 1 [file Table_1.docx]

Supplementary Material

# Supplementary Figures and Tables

## Supplementary Figures


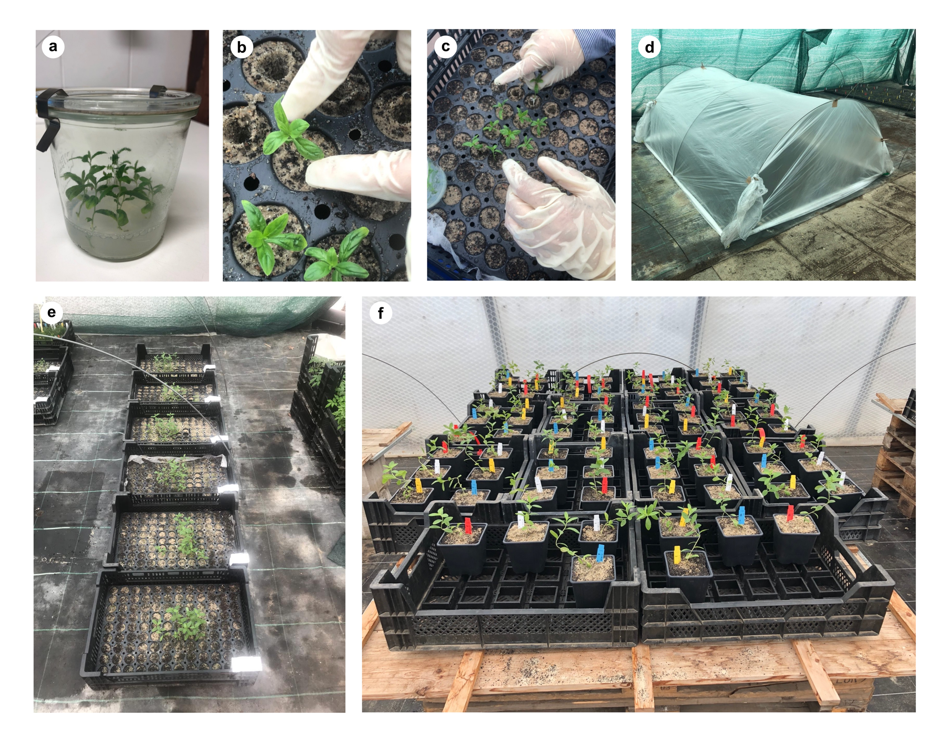


**Supplementary Figure 1.** Greenhouse experiment. Pictures of the different steps of the set-up. (a) Plants were grown for 10 days in MS^mod^ medium prior to the transfer. (b, c) Transfer of the rooted plants to a tray for acclimatisation. (d) A tent was built over the boxes for acclimatisation. (e) After 3 weeks of acclimation, the plants were left out of the tent for one week prior to the transfer to individual pots. (f) Plants transplanted to 1 L individual pots and randomised for the experiment.


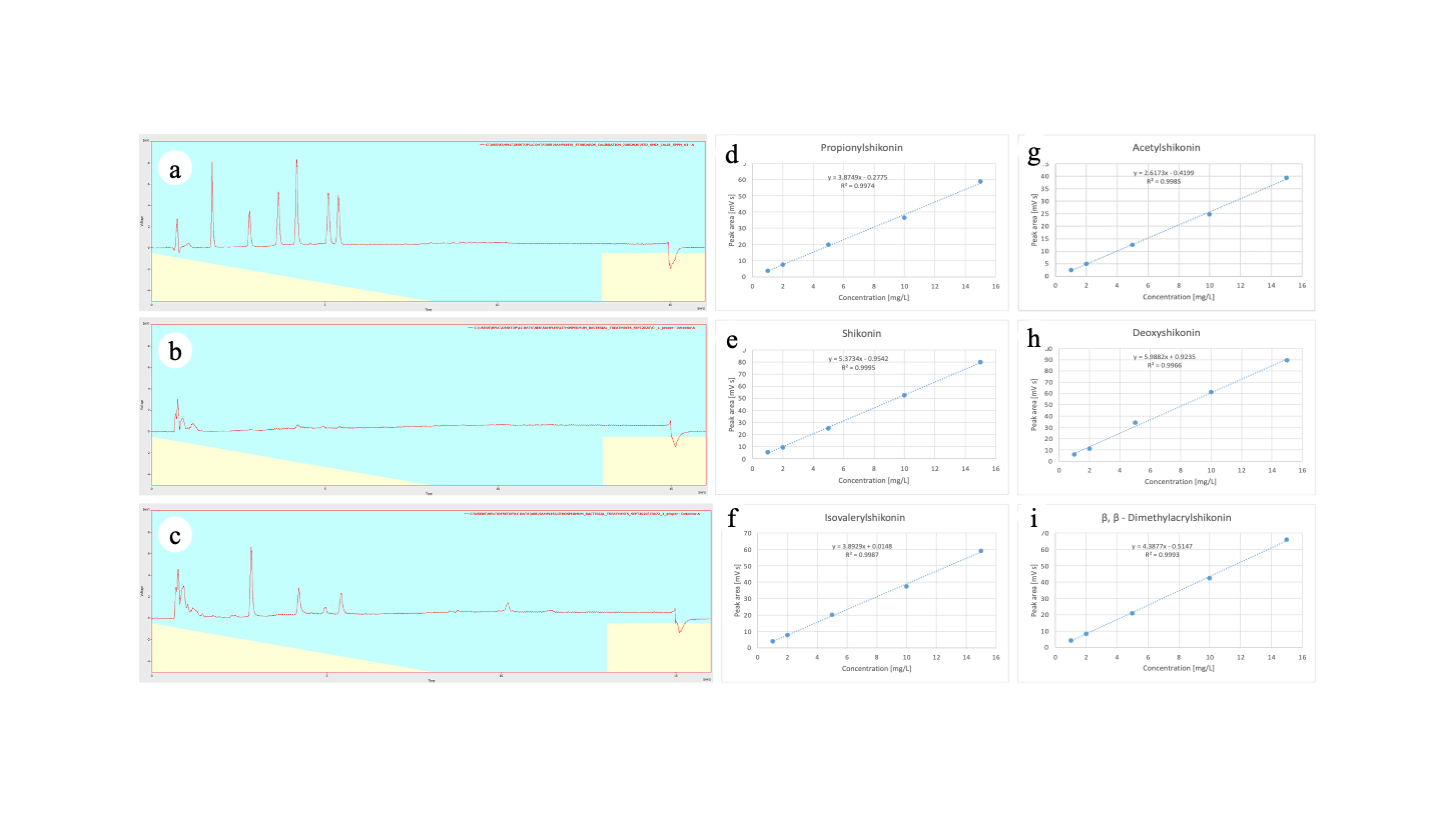


**Supplementary Figure 2.** Examples of HPLC chromatograms: a) Chromatogram of a mixture containing standards of shikonin (1.7 min), acetylshikonin (2.8 min), propionylshikonin (3.7 min), deoxyshikonin (4.2 min), β, β – dimethylacrylshikonin (5.1 min) and isovalerylshikonin (5.4 min), all compounds are present in a concentration of 5 mg/L. Absorbance measured at 520 nm. b) Chromatogram of a negative control sample, showing no detectable concentration of A/S. c) Chromatogram of a sample of *Lithospermum officinale* roots inoculated with *Chitinophaga sp.* R-73072. Panels d to i: Calibration curves of the different standards used for the HPLC-DAD.


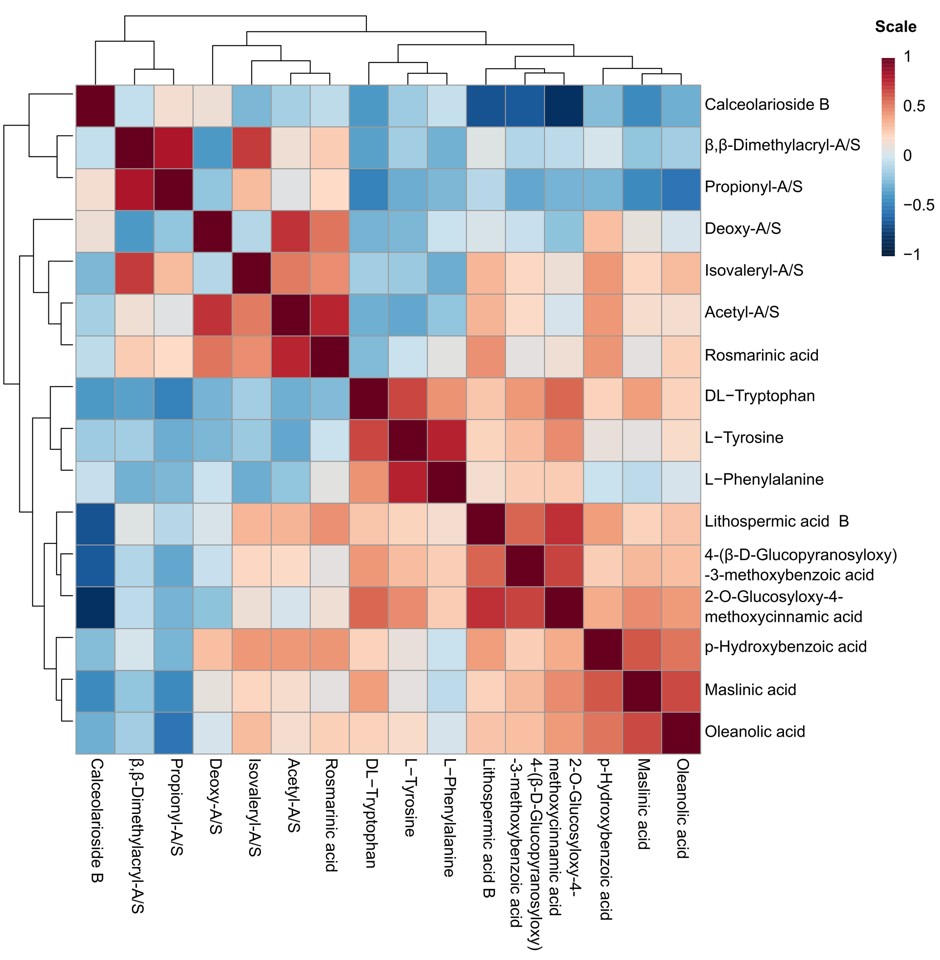
**Supplementary Figure 3.** Correlations of compounds detected and identified by UHPLC-HRMS. Treatments include non-inoculated control and six bacterial treatments (6 biological replicates per treatment). The matrix of normalized peak intensities was analysed with the online server MetaboAnalyst 5 using the module univariate analysis (correlation heatmaps). Spearman rank correlation was used to measure the distance and clustering of features. Metabolite intensity values were auto-scaled using standard settings (range from 1 to -1) for visual representation in the heatmap.

## Supplementary Tables

**Supplementary Table S1.** Nutrient medium composition comparison. MS^mod^, MS^mod rooting^ and MSR^mod^ against the original MS (Murashige and Skoog, 1962) and MSR media (Declerck *et al*., 1998) composition.

| **Chemicals** | **MS** | **MS^mod^** | **MS^mod rooting^** | **MSR** | **MSR^mod^** |
| --- | --- | --- | --- | --- | --- |
|  | (=mg/L Medium) | | | | |
| **Macroelements** | | | | | |
| KNO_3_ | 1900 | 1900 | 950 | 76 | 76 |
| Ca(NO_3_)_2_ |  |  |  |  |  |
| NH_4_NO_3_ | 1650 | 1650 | 825 |  |  |
| MgSO_4_ x 7 H_2_O | 370 | 370 | 185 | 739 | 739 |
| KH_2_PO_4_ | 170 | 170 | 85 | 4.1 | 4.1 |
| KCl |  |  |  | 65 | 65 |
| CaCl_2_ x 2 H_2_O | 440 | 440 | 220 |  |  |
| Ca(NO3)_2_ x 4 H_2_O |  |  |  | 359 | 359 |
| **Iron** | | | | | |
| NaFeEDTA | 36.7 | 37.5 | 37.5 | 8.12 | 8.12 |
| **Microelements** | | | | | |
| MnCl_2_ x 4 H_2_O |  |  |  | 2,45 | 2,45 |
| MnSO_4_ x H_2_O | 17 | 17 | 17 |  |  |
| H_3_BO_3_ | 6.2 | 6.2 | 6.2 | 1.86 | 1.86 |
| ZnSO_4_ x 7 H_2_O | 8.6 | 8.6 | 8.6 | 0.29 | 0.29 |
| Na_2_MoO_4_ x 2 H_2_O | 0.25 | 0.25 | 0.25 | 0.0024 | 0.0024 |
| CuSO_4_ x 5 H_2_O | 0.025 | 0.025 | 0.025 | 0.24 | 0.24 |
| CoCl_2_ x 6 H_2_O | 0.025 | 0.025 | 0.025 |  |  |
| KI | 0.83 | 0.83 | 0.83 |  |  |
| (NH_4_)_6_Mo_7_O_24_ x 4 H_2_O |  |  |  | 0.035 |  |
| **Vitamins** | | | | | |
| Thiamin HCl | 0.1 | 0.1 | 0.1 | 1 | 1 |
| Nicotin acid | 0.5 | 0.5 | 0,5 | 1 | 1 |
| Pyridoxine | 0.5 | 0.5 | 0.5 | 0.9 | 0.9 |
| Glycine | 2 | 2 | 2 |  |  |
| CaPanthotenate |  |  |  | 0.9 | 0.9 |
| Biotin |  |  |  | 0.0009 | 0.0009 |
| Cyanocobalamine |  |  |  | 0.4 | 0.4 |
| myo-Inositol | 100 | 100 | 100 |  |  |
| **Sugars (g/L)** | | | | | |
| Saccharose | 30 | 10 | 10 | 10 |  |
| **Agar (g/L)** | | | | | |
| Phytoagar | 6 | 6 | 6 |  |  |
| Gelrite |  |  |  | 3 | 3 |
| **pH** | | | | | |
|  | 5.8 | 5.8 | 5.8 | 5.5 | 5.5 |

**Supplementary Table S2.** Primers used for qPCR.

| **Target gene** | **Primer** | **Sequence (5′ - 3′)** | **Reference** |
| --- | --- | --- | --- |
| Housekeeping gene for Actin-7; ACT7. | LeACT7_Fw | GCCTTTATTGTCTGCATCACC | Ueoka *et al*., 2020 |
| Housekeeping gene for Actin-7; ACT7. | LeACT7_Rv | GGGGAAAATAGAAAGCCAAAAC | Ueoka *et al*., 2020 |
| Housekeeping gene for glyceraldehyde 3-phosphate dehydrogenase; GAPDH. | GAPDH_Fw | ACCGTCCACTCCATTACCG | Wu *et al*., 2009 |
| Housekeeping gene for glyceraldehyde 3-phosphate dehydrogenase; GAPDH. | GAPDH_Rv | ATGAGGCAGCCCTTCCACC | Wu *et al*., 2009 |
| Expession of 4-hydroxybenzoate geranyltransferase; PGT (A/S precursor) | PGT_Fw | CTCTTAGGCTCCTCTGCT | Wu *et al*., 2009 |
| Expession of 4-hydroxybenzoate geranyltransferase; PGT (A/S precursor) | PGT_Rv | CGTCGTCCACCTTATCTT | Wu *et al*., 2009 |

**Supplementary Table S3.** The phenotypic effect of bacterial inoculation on the plant roots. Bacteria in bold were selected for further assays.

| **Controls** | | | | | |
| --- | --- | --- | --- | --- | --- |
| **Test** |  | **Effect** | **Test** |  | **Effect** |
| Negative control | PBS inoculation | Normal root growth + no pigmentation | Positive  control | MeJA inoculation | Root inhibition + pigmentation (purple) |
| **Group 1: No root growth inhibition + no pigmentation** | | | | | |
| **Strain** | **Identification** | **Pigmentation** | **Strain** | **Identification** | **Pigmentation** |
| R-71825 | *Roseomonas* sp. | None | R-72208 | *Neorhizobium* sp. | None |
| R-71842 | *Pseudomonas* sp. | None | R-72249 | *Pedobacter* sp. | None |
| R-71893 | *Bacillus* sp. | None | R-72369 | *Methylopila* sp. | None |
| R-71903 | *Rhizobium* sp. | None | R-72406 | *Stenotrophomonas* sp. | None |
| R-71954 | *Rhizobium* sp. | None | **R-72433** | ***Rhizobium* sp.** | None |
| R-71986 | *Pseudoxanthomonas* sp. | None | R-72446 | *Variovorax* sp. | None |
| R-72066 | *Rhizobium* sp. | None | R-72456 | *Rhizobium* sp. | None |
| R-72074 | *Pseudomonas* sp. | None | R-72492 | *Bacillus* sp. | None |
| R-72135 | *Pseudomona*s sp. | None | R-72495 | *Variovorax* sp. | None |
| R-72139 | *Methylobacterium* sp. | None | R-72498 | *Pantoea* sp. | None |
| R-72151 | *Luteibacter* sp. | None | R-72500 | *Rhizobium* sp. | None |
| R-72153 | *Variovorax* sp. | None | R-72501 | I*nquilinus* sp. | None |
| R-72160 | *Rhizobium* sp. | None | R-72562 | *Kocuria* sp. | None |
| R-72164 | *Pseudomonas* sp. | None | R-72591 | *Sphingobium* sp. | None |
| R-72172 | *Pseudomonas* sp. | None | R-72597 | *Tardiphaga* sp. | None |
| R-72191 | *Olivibacter* sp. | None | R-73343 | *Acidovorax* sp. | None |
| **Group 2: No root growth inhibition + pigmentation** | | | | | |
| **Strain** | **Identification** | **Pigmentation** | **Strain** | **Identification** | **Pigmentation** |
| **R-71838** | ***Pseudomonas* sp.** | brownish | R-72599 | *Pseudomonas* sp. | brownish/black |
| **R-71875** | ***Brevibacterium* sp.** | brownish/black | R-72634 | *Bacillus* sp. | brownish/black |
| R-72115 | *Shinella* sp. | brownish/black | **R-73072** | ***Chitinophaga* sp.** | black |
| **R-72269** | ***Chitinophaga* sp.** | brownish/purple | **R-73098** | ***Xanthomonas* sp.** | brownish/black |
| R-72393 | *Pedobacter* sp. | Black | R-73111 | *Phyllobacterium* sp. | brownish/black |
| R-72401 | *Shinella* sp. | brownish/black | R-73117 | *Rhizobium* sp. | brownish/black/purple |
| R-72464 | *Xanthomonas* sp. | brownish/black |  |  |  |
| **Group 3: Root growth inhibition + pigmentation** | | | | | |
| **Strain** | **Identification** | **Pigmentation** | **Strain** | **Identification** | **Pigmentation** |
| R-71941 | *Tsukamurella* sp. | brownish/black | R-72102 | *Pseudomonas* sp. | brownish |
| R-72008 | *Pseudomonas* sp. | brownish | R-72251 | *Xanthomonas* sp. | brownish/black |

Supplementary Table S4. Shoot length of the different treatments of plants grown under polytunnel conditions (10 biological replicates per treatment). Data are expressed in cm and were compared with one-factor ANOVA (p= 0.01669< 0.05) followed by a Newman-Keuls test. Means associated with the same letter are not considered to be significantly different.

| **Treatments** | **Means** |
| --- | --- |
| *Chitinophaga* sp. R-73072 | 21.97 ± 2.49 a |
| Pseudomonas sp. R-71838 | 21.02 ± 3.42 a |
| *Rhizobium* sp. R-72433 | 20.75 ± 3.32 a |
| *Brevibacterium* sp. R-71875 | 20.63 ± 3.92 a |
| *Xanthomonas* sp. R-73098 | 20.50 ± 1.97 a |
| *Chitinophaga* sp. R-72269 | 20.38 ± 2.62 a |
| Negative control (MSR^mod^) | 16.90 ± 1.82 b |

Supplementary Table S5. Plant weights (root and shoot) of the different treatments of plants grown under polytunnel conditions (10 biological replicates per treatment). Data of root weight were compared with one-factor ANOVA followed by a Newman-Keuls test. Data of shoot weight were compared with Kruskal-Wallis followed by a Dunn test. Means associated with the same letter are not considered to be significantly different.

| **Root dry weight (g)** | | **Shoot dry weight (g)** | |
| --- | --- | --- | --- |
| **p-value 0.0111** | | **p-value 0.005962** | |
| **Treatments** | **Means** | **Treatments** | **Means** |
| R-72269 | 1.07 ± 0.22 a | R-71838 | 0.55 ± 0.28 a |
| R-73072 | 1.02 ± 0.26 ab | R-73072 | 0.50 ± 0.10 a |
| R-71838 | 1.01 ± 0.34 ab | R-71875 | 0.47 ± 0.16 a |
| R-72433 | 0.86 ± 0.34 ab | R-72269 | 0.47 ± 0.07 a |
| R-71875 | 0.84 ± 0.38 ab | R-72433 | 0.44 ± 0.15 ab |
| R-73098 | 0.73 ± 0.22 ab | R-73098 | 0.37 ± 0.10 ab |
| Negative control | 0.65 ± 0. 20 b | Negative control | 0.32 ± 0.07 b |

**Supplementary Table S6.** Identified metabolites within all plant root samples, inoculated with different bacterial treatments and grown *in vitro*. Identification of compounds detected by UHPLC-HRMS was performed using the Compound Discoverer software.

| **Compound Identification** | **Chemical Class** | **Molecular Formula** | **Molecular Weight (g/mol)** | **MS/MS fragmentation match (%)** |
| --- | --- | --- | --- | --- |
| Acetyl-A/S | Naphthoquinone | C_18_H_18_O_6_ | 330.3 | 100.0 |
| Shikonin | Naphthoquinone | C_16_H_16_O_5_ | 288.29 | 100.0 |
| Isovaleryl-A/S | Naphthoquinone | C_21_H_24_O_6_ | 372.4 | 100.0 |
| β,β-Dimethylacryl-A/S | Naphthoquinone | C_21_H_22_O_6_ | 370.4 | 100.0 |
| Deoxy-A/S | Naphthoquinone | C_16_H_16_O_4_ | 272.29 | 100.0 |
| Propionyl-A/S | Naphthoquinone | C_19_H_20_O_6_ | 344.4 | 100.0 |
| Rutin | Flavonol glycoside | C_27_H_30_O_16_ | 610.5 | 94.8 |
| 4-(β-D-Glucopyranosyloxy)-3-methoxybenzoic acid | Hydroxycinnamic acid | C_14_H_18_O_9_ | 330.29 | 97.8 |
| Calceolarioside B | Hydroxycinnamic acid | C_23_H_26_O_11_ | 478.4 | 93.5 |
| 2-O-Glucosyloxy-4-methoxycinnamic acid | Hydroxycinnamic acid | C_16_H_20_O_9_ | 356.32 | 46.5 |
| p-Hydroxybenzoic acid | Phenolic acid | C_7_H_6_O_3_ | 138.12 | 53.4 |
| Lithospermic acid B | Polyphenol | C_36_H_30_O_16_ | 718.6 | 92.5 |
| Rosmarinic acid | Polyphenol | C_18_H_16_O_8_ | 360.3 | 95.2 |
| Adenosine | Ribonucleoside | C_10_H_13_N_5_O_4_ | 267.24 | 99.0 |
| Maslinic acid | Triterpene | C_30_H_48_O_4_ | 472.7 | 89.6 |
| Oleanolic acid | Triterpene | C_30_H_48_O_3_ | 456.7 | 95.3 |
| Thiamine | Vitamin | C_12_H_17_N_4_OS^+^ | 265.36 | 98.0 |
| L-Asparagine | Amino acid | C_4_H_8_N_2_O_3_ | 132.12 | 91.1 |
| L-Tyrosine | Amino acid | C_9_H_11_NO_3_ | 181.19 | NA |
| L-Histidine | Amino acid | C_6_H_9_N_3_O_2_ | 155.15 | 83.3 |
| L-Phenylalanine | Amino acid | C_9_H_11_NO_2_ | 165.19 | 55.9 |
| DL-Glutamine | Amino acid | C_5_H_10_N_2_O_3_ | 146.14 | 90.7 |
| DL-Tryptophan | Amino acid | C_11_H_12_N_2_O_2_ | 204.22 | 90.7 |
| α-Linolenic acid | Fatty acid | C_18_H_30_O_2_ | 278.4 | 91.0 |
| 12-Oxo-phytodienoic acid | Fatty acid | C_18_H_28_O_3_ | 292.4 | 86.0 |

Supplementary Table S7. Expression of key gene PGT (core A/S pathway), as quantified by qPCR. Data were compared with one-factor ANOVA followed by a t-test. Inoculated (bacteria) and non-inoculated plant treatments (3 biological replicates per treatment) were statistically compared (p-value = 0.007) using qbase+ v 3.2.

| **Comparison** | **Ratio** | **Significance** |
| --- | --- | --- |
| R-72433/Control | 1.78 | Yes |
| R-73072/Control | 2.41 | Yes |
| R-73072/R-72433 | 1.35 | No |
